# Membrane fluidification by ethanol stress activates unfolded protein response in yeasts

**DOI:** 10.1111/1751-7915.13032

**Published:** 2018-02-22

**Authors:** Elisabet Navarro‐Tapia, Amparo Querol, Roberto Pérez‐Torrado

**Affiliations:** ^1^ Instituto de Agroquímica y Tecnología de los Alimentos IATA‐CSIC E‐46980 Paterna Valencia Spain

## Abstract

The toxic effect of ethanol is one of the most important handicaps for many biotechnological applications of yeasts, such as bioethanol production. Elucidation of ethanol stress response will help to improve yeast performance in biotechnological processes. In the yeast *Saccharomyces cerevisiae,* ethanol stress has been recently described as an activator of the unfolded protein response (UPR), a conserved intracellular signalling pathway that regulates the transcription of ER homoeostasis‐related genes. However, the signal and activation mechanism has not yet been unravelled. Here, we studied UPR's activation after ethanol stress and observed the upregulation of the key target genes, like *INO1*, involved in lipid metabolism. We found that inositol content influenced UPR activation after ethanol stress and we observed significant changes in lipid composition, which correlate with a major membrane fluidity alteration by this amphipathic molecule. Then, we explored the hypothesis that membrane fluidity changes cause UPR activation upon ethanol stress by studying UPR response against fluidification or rigidification agents and by studying a mutant, erg2, with altered membrane fluidity. The results suggest that the membrane fluidification effects of ethanol and other agents are the signal for UPR activation, a mechanism that has been proposed in higher eukaryotes.

## Introduction

Yeasts are key organisms involved in a myriad of biotechnological applications, such as the production of alcoholic drinks or fuels as bioethanol. Crabtree‐positive yeast species, like *Saccharomyces cerevisiae*, growing on high sugar concentrations produce high levels of ethanol, a toxic compound which, paradoxically, severely affects the physiology of yeasts at several levels (Alexandre *et al*., 2001; Yang *et al*., 2012; Ma *et al*., 2013) and limits their biotechnological potential. Ethanol is a small two‐carbon alcohol which, given its short alkene chain and the hydroxyl group, is soluble in both aqueous and lipid environments and can pass to cells through the plasmatic membrane by producing an increase in membrane fluidity (Jones and Greenfield, 1987; Lloyd *et al*., 1993). This fluidity increase causes loss of membrane integrity and favours permeability (Marza *et al*., 2002). Ethanol also alters the mitochondrial structure, lowers ATP levels and respiratory rates and favours the generation of acetaldehyde and reactive oxygen species (ROS), which ultimately produce lipid peroxidation, DNA damage, oxidative stress and, consequently, reduce cell viability (Alexandre *et al*., 2001; Yang *et al*., 2012; Ma *et al*., 2013).

Several studies have offered some clues about the molecular basis that underlies yeast resistance and response to ethanol stress (Ma and Liu, 2010; Stanley *et al*., 2010). There is a well‐established correlation between ethanol resistance and the increase in the degree of fatty acids unsaturation of membrane lipids to antagonize the fluidification effect (Alexandre *et al*., 1994; You *et al*., 2003). Indeed, it has been shown that supplementation with palmitoleic and oleic acid, two essential unsaturated fatty acids present in yeast membranes, reduces cell viability loss upon ethanol stress (Thomas *et al*., 1978; You *et al*., 2003). Other studies have shown that strains with higher levels of unsaturated fatty acids are more tolerant to the effects of ethanol on the cellular membrane (Chi and Arneborg, 1999; Aguilera *et al*., 2006). This effect has also been described in ethanol‐resistant bacteria (Kinji, 1974; Ingram, 1990), while accumulation of other lipids, such as ergosterol, has been related to ethanol resistance due to an increased stability of membranes (Shobayashi *et al*., 2005; Aguilera *et al*., 2006).

Unlike cell wall or osmotic stress, ethanol stress does not seem to involve a specific response pathway, but has been related to existing signalling pathways (Takemura *et al*., 2004). It has been shown that ethanol activates the unfolded protein response (UPR) (Brown *et al*., 2013; Miyagawa *et al*., 2014; Navarro‐Tapia *et al*., 2016), which is conserved across eukaryotes to restore and enhance the secretory and protein‐folding capacity of the endoplasmic reticulum (ER) (Mori, 2000; Ron and Walter, 2007; Walter and Ron, 2011). The UPR pathway in yeast cells includes an ER membrane sensor that undergoes oligomerization and autophosphorylation after binding unfolded proteins. This sensor, known as Ire1 (inositol responsive element 1), acquires endoribonuclease activity when activated, and then catalyses the splicing of Hac1, a transcription factor that activates hundreds of genes that restore the normal ER function (Cox and Walter, 1996; Sidrauski and Walter, 1997). Although the conventional signal that triggers Ire1 activation is accumulation of unfolded proteins in the ER, a drastic drop in inositol content has been shown to also activate the UPR pathway (Cox *et al*., 1997; Promlek *et al*., 2011). The rationale to explain this mechanism of activation lies in the fact that *INO1*, which encodes an essential enzyme for inositol biosynthesis, is activated to restore lipid levels (Greenberg and Lopes, 1996).

This work focused on elucidating the molecular basis of the mechanism that underlies UPR activation in response to ethanol stress. Previous studies have suggested that unfolded protein accumulation or inositol depletion does not seem to be the signal for UPR activation after ethanol stress (Navarro‐Tapia *et al*., 2017). Here, we hypothesized that membrane fluidity changes are the origin of UPR activation by ethanol stress. First, we observed that inositol influences UPR activation after ethanol stress. A change in lipid composition that counteracts the fluidification of membranes by ethanol was also observed. Finally, we studied the UPR response after adding fluidification or rigidification agents, and the UPR response in the erg2 mutant with altered membrane fluidity (Sharma, 2006). The results suggested that UPR was activated due to the membrane fluidification effects of ethanol and other agents.

## Results

### Ethanol stress activates UPR independently of unfolded protein accumulation

**Figure 1:**
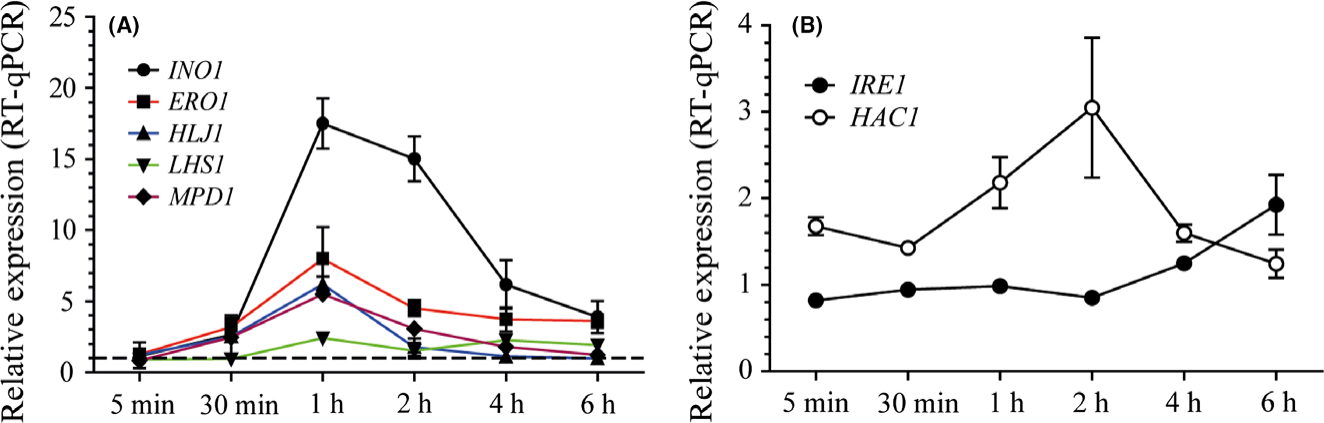
Relative expression of UPR target genes after ethanol exposure. Cells were exposed to 8% ethanol, and samples were obtained at different time points. Each data point was referred to a control experiment exposed to 0% of ethanol and to time point 0. The mRNA levels of both the target genes (A) and the UPR central components (B) were determined by qPCR after normalization with two constitutive control genes. The results represent the average and standard deviation of three independent biological replicates.

To gain insights into the mechanism that activates UPR in response to ethanol stress, we focused on the key target genes that have been previously characterized for their activation through this pathway after ER stress. We studied the mRNA levels of a gene involved in inositol metabolism, *INO1,* and four genes involved in protein folding, *ERO1*,* LHS1*,* HLJ1* and *MPD1*, after exposing cells to physiological levels of ethanol (8%). The results were compared to the data obtained from cells maintained under the same conditions, but without adding ethanol. We also studied UPR pathway genes *HAC1* and *IRE1*, which were activated in a positive feedback loop. The results (Fig. 1) revealed gene activations after a 30‐minute exposure and showed maximal activations 1 h after ethanol stress. Interestingly, the gene that played a role in inositol biosynthesis, *INO1,* was the most activated gene (Fig. 1A), which suggests that lipid metabolism plays a determinant role in the response to ethanol stress. UPR sensor *IRE1* (Fig. 1B) showed a slight upregulated expression after 6 h (*t*‐test, *P* < 0.05).

Activation of key target genes agrees with changes observed in previous studies upon RE stress (Travers *et al*., 2000; Kimata *et al*., 2006). Some of these genes have also been observed in other transcriptomic studies related to ethanol stress, such as *INO1* (Dinh *et al*., 2009; Lewis *et al*., 2014; Navarro‐Tapia *et al*., 2016), *ERO1* (Alexandre *et al*., 2001; Dinh *et al*., 2009; Lewis *et al*., 2014), *LHS1* (Alexandre *et al*., 2001; Lewis *et al*., 2014) and *HAC1* (Navarro‐Tapia *et al*., 2016). The strong *INO1* activation that we observed has been described in other studies (Dinh *et al*., 2009; Lewis *et al*., 2014; Navarro‐Tapia *et al*., 2016). The late and slight *IRE1* activation (*t*‐test, *P *<0.05) was not observed in RE or ethanol stress transcriptomic studies.

**Figure 2:**
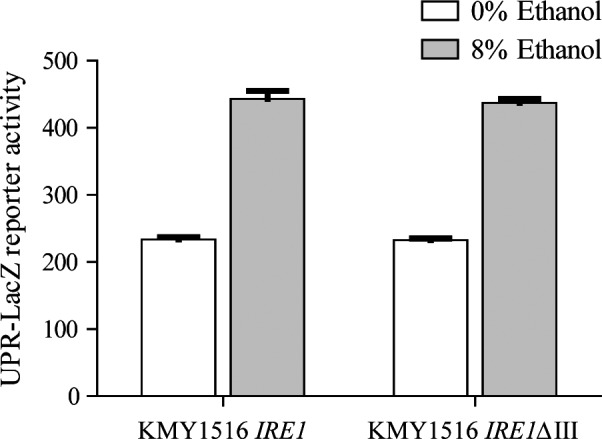
UPR activation after ethanol stress in the strains that contained wild‐type Ire1 or the Ire1ΔIII mutant. The cells that contained wild‐type Ire1 or Ire1ΔIII were exposed to 8% ethanol, and the samples in the medium with 90 μM inositol were obtained after 6 h. UPR activation was determined by measuring β‐galactosidase activity using the UPR‐LacZ reporter. The results represent the average and standard deviation of three independent biological replicates.

In our previous study (Navarro‐Tapia *et al*., 2017), we suggested that ethanol stress did not promote unfolded protein accumulation in the ER. To gain insights as to whether unfolded protein accumulation or membrane alterations are the signals that activate UPR in response to ethanol stress, we tested UPR activation in the presence of the Ire1ΔIII mutant described by (Promlek *et al*., 2011). This is a version of the Ire1 sensor without the domain that binds unfolded proteins, which is activated only after membrane alterations. Here, we showed that the strain that harboured the wild‐type version of Ire1 activated the UPR‐LacZ reporter after 8% ethanol (Fig. 2). Interestingly, the strain that harboured the IreΔIII mutant also activated the UPR after ethanol stress, which confirmed our hypothesis that the membrane alterations produced by this toxic were the main signal involved in UPR firing.

### Inositol content influences UPR activation

**Figure 3:**
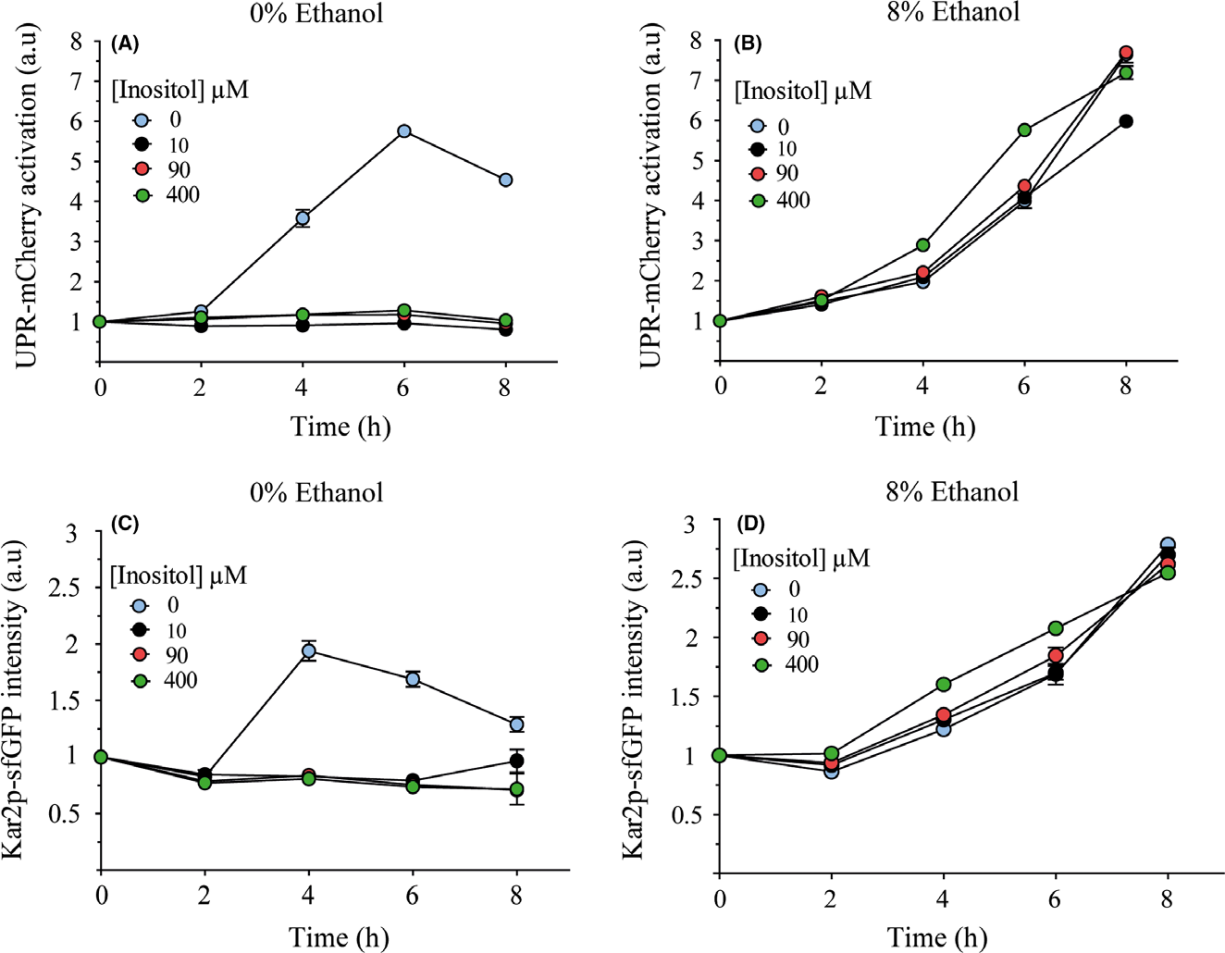
UPR activation dependence on the presence of ethanol stress and inositol content. Cells were exposed to different levels of inositol content (0, 10, 90 and 400 μM) alone (A, C) or with 8% ethanol (B, D). Samples were obtained at different time points. The fluorescence of the UPR‐mCherry (A, B) or Kar2‐sfGPF (C, D) reporters was measured by flow cytometry. The results refer to time point 0 for each condition and reporter and represent the average and standard deviation of three independent biological replicates.

As absence of inositol can activate UPR signalling (Cox *et al*., 1997; Promlek *et al*., 2011), we studied if inositol content could influence UPR activation after ethanol stress. We determined the level of two fluorescent reporters, UPR‐cherry and Kar2p‐sfGFP, integrated into the genome of a yeast strain that responds to this pathway. The results (Fig. 3) showed the previously described UPR activation, where inositol was absent and no UPR activation took place with the presence of 10, 90 or 400 μM of inositol in media (Fig. 3A and C). Furthermore, both reporters were activated after 8% ethanol (Fig. 3B and D) and also under different inositol content conditions. Activation became significantly greater at time points 4 and 6 h (*t*‐test, *P* < 0.05), when 400 μM of inositol was present, than under other conditions, which suggests that certain levels of phosphatidylinositol (PI) membrane content, which participate in the increase in membrane fluidity (de Kroon *et al*., 2013), can influence UPR activation after ethanol stress. These results also suggest that ethanol does not seem to produce inositol depletion, which might explain UPR activation. The absence of inositol and ethanol stress had no additive effect on UPR activation (compare 3A with 3B, and 3C with 3D). In fact they both showed even significantly lower levels (*t*‐test, *P* < 0.05 when both conditions were present) at time points 4 and 6 h for UPR‐mCherry, and at time point 4 h for the Kar2p‐sfGFP reporter, which implies that different mechanisms could be implicated in each activation type. Regarding growth (Fig. S1), ethanol stress significantly reduced yeast growth, which could barely duplicate biomass after 8 h. The absence of inositol also affected growth when no ethanol was present.

### Ethanol stress induces membrane changes in membrane fluidity regulatory lipids

**Figure 4:**
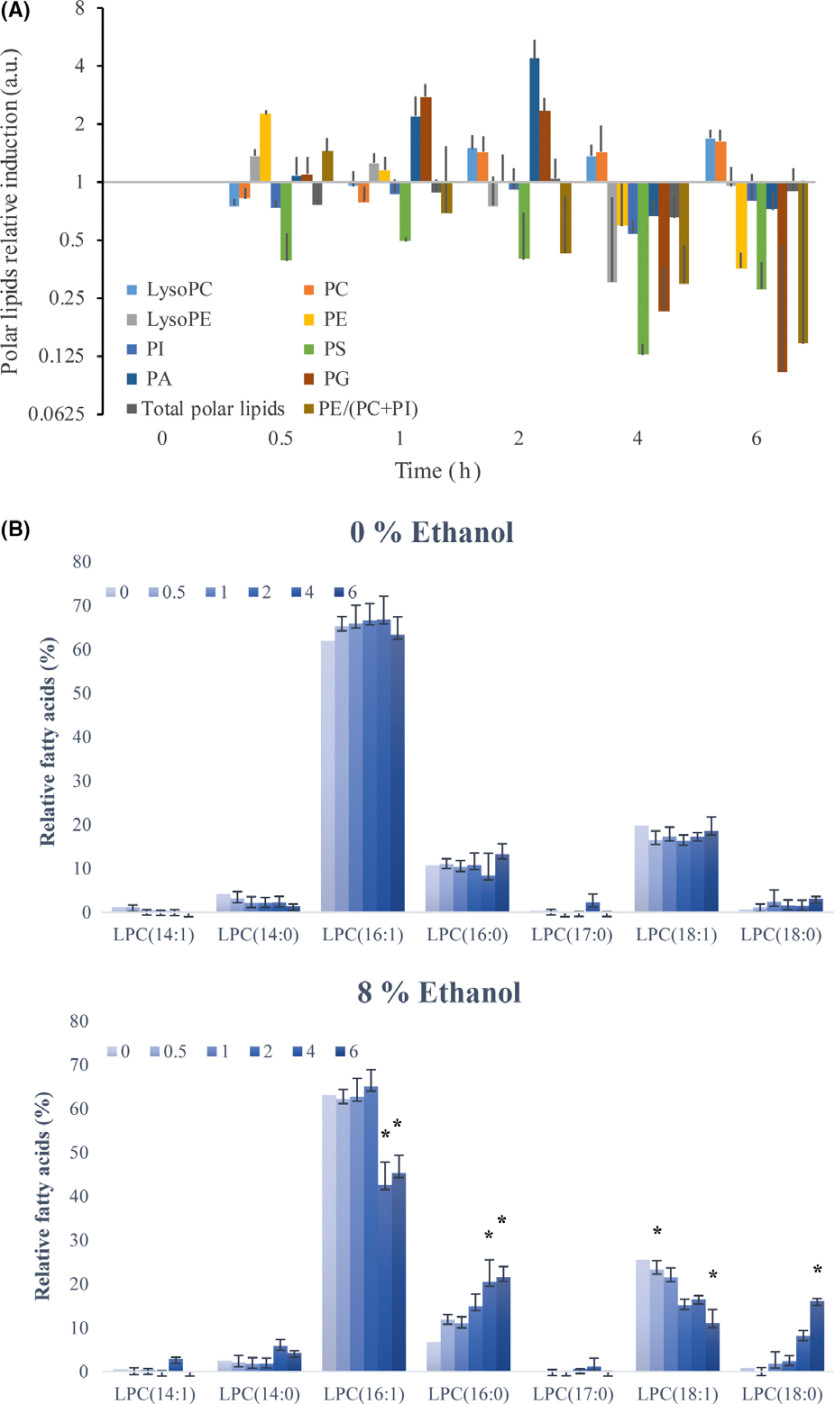
Changes in membrane fluidity regulatory lipids after ethanol stress. Cells were exposed to 8% ethanol, and samples were obtained at different time points. (A) Polar lipid levels were determined by ESI‐MS/MS with restrictive quality controls. The quantified compounds are phosphatidic acid (PA), phosphatidylethanolamine lipids (PE), phosphatidylcholine (PC), LysoPC, phosphatidylserine (PS), LysoPE, phosphatidylglycerol (PG) and phosphatidylinositol (PI). The results are normalized with the levels of the samples obtained in parallel in a medium with 0% ethanol and refer to time point 0 for each lipid. (B) The relative levels of the different fatty acids were also represented in both conditions. The results represent the average and standard deviation of three independent biological replicates. Significant differences (*P* < 0.05) between conditions (with or without 8% ethanol) are indicated by an asterisk.

Increases in membrane fluidity feature among the most important effects of ethanol on yeast cells (Marza *et al*., 2002). Here, we intended to study if cells under our conditions, where UPR was activated, could change membrane composition in polar lipids in response to ethanol membrane fluidification. Cells were exposed to 8% ethanol, and samples were obtained at different time points. Polar lipid levels were determined by ESI‐MS/MS with quality controls. The results were normalized with the levels of the samples obtained in parallel in a medium with 0% ethanol. The results showed major changes in different polar lipids, although the total amount remained unaltered throughout the experiment (Fig. 4A). We observed an increased phosphatidic acid (PA) lipid species content, which reached a maximal level 2 h after ethanol stress. After the initial accumulation, phosphatidylethanolamine lipids (PE) lowered with time. We observed a slight increase in phosphatidylcholine (PC) and LysoPC, as well as significant decreases (*t*‐test, *P* < 0.05) in phosphatidylserine (PS) and LysoPE. Like PA, phosphatidylglycerol (PG) increased, but decreased at the last time points. No significant variation (*t*‐test, *P* < 0.05) in the total phosphatidylinositol (PI) levels was observed. Since an inverse correlation between the PE/(PC + PI) cellular levels and membrane fluidity is well‐established (de Kroon *et al*., 2013), we calculated this parameter. As observed, a reduction pattern appeared after ethanol exposure, which confirmed a cellular response against the ethanol fluidification effect. We also studied the unsaturation levels in the principal acyl side‐chains (Fig. 4B) as a decrease in the degree of unsaturation has been described as a conserved cellular response to reduce membrane fluidity (Ballweg and Ernst, 2016; Ernst *et al*., 2016). Under our conditions, we observed a significant decrease (*t*‐test, *P* < 0.05) in the mono‐unsaturated acyl chains (C16:1 and 18:1), as well as a significant increase (*t*‐test, *P* < 0.05) in saturated fatty acids (C16:0 and 18:0) at different time points in the PC species. Similar results were obtained in the PE species (results not shown). Altogether, these results suggest that cells alter membrane lipid composition by reversing the fluidification effects of physiological levels of ethanol.

### Membrane fluidification agents activate UPR

**Figure 5:**
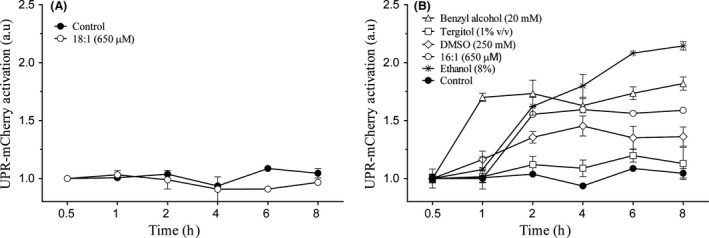
Influence of membrane fluidification agents upon UPR activation after ethanol exposure. Cells were exposed to a different membrane rigidification (A) of fluidification agents (B) and samples were obtained at different time points. UPR‐mCherry reporter fluorescence was measured by flow cytometry. The results refer to time point 0 for each condition and represent the average and standard deviation of three independent biological replicates.

**Figure 6:**
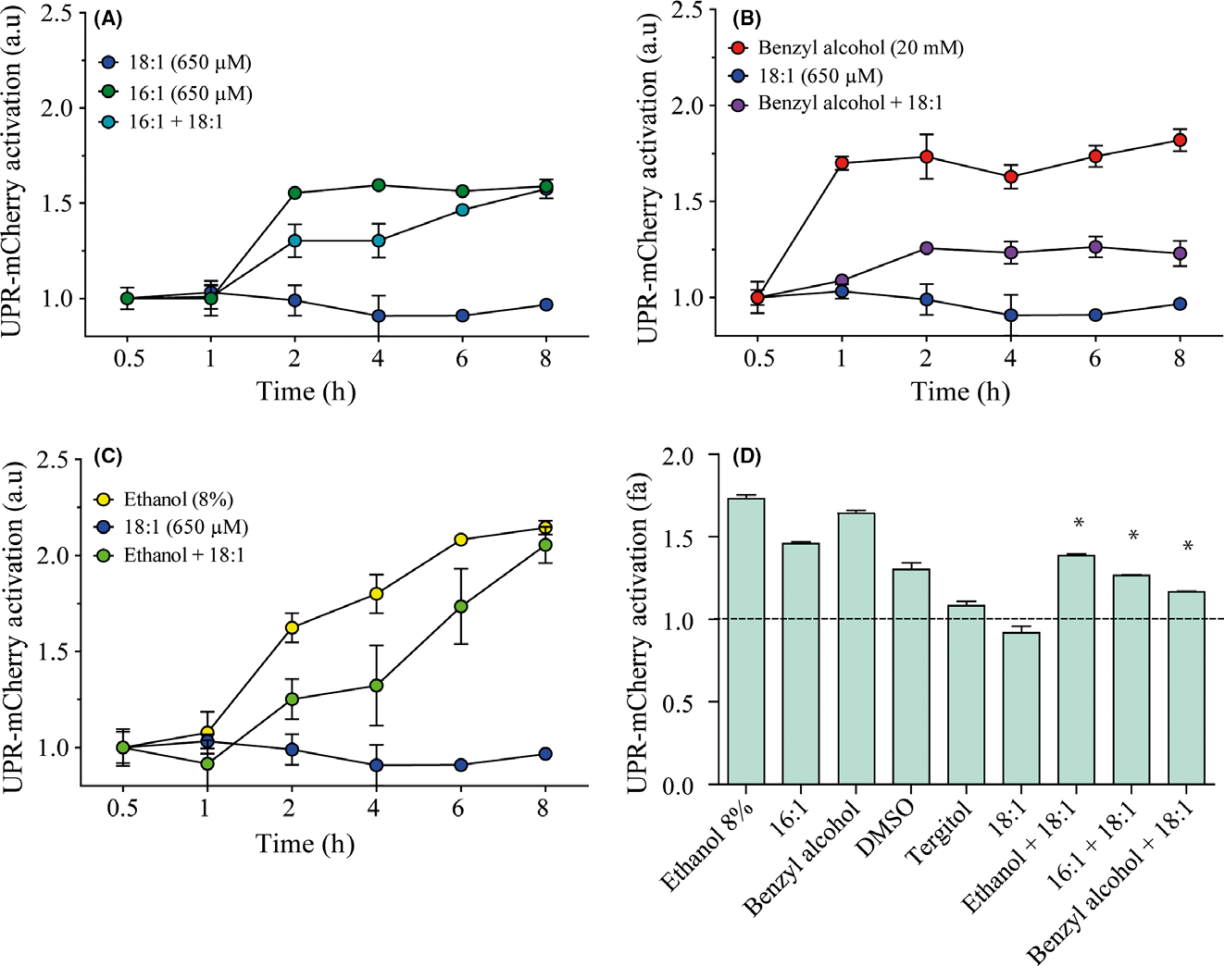
Effect of rigidification agent oleic acid (18:1) upon UPR activation produced by fluidification agents. Cells were exposed to different membrane fluidification agents, palmitoleic acid (16:1) (A), benzyl alcohol (B) and ethanol (C), with or without oleic acid (18:1). Samples were obtained at different time points. UPR‐mCherry reporter fluorescence was measured by flow cytometry. The results refer to time point 0 for each condition and represent the average and standard deviation of three independent biological replicates. (D) Quantification of the different activation levels was performed by determining the area under the curve. The significant differences (*P* < 0.05) between conditions, with or without 18:1, are indicated by an asterisk.

To evaluate whether different compounds with effects on membrane fluidity other than ethanol can also activate UPR signalling, we studied the UPR‐mCherry activation reporter in response to their presence (Fig. 5). First, we observed the response against oleic acid (C18:1), which has been described as a membrane fluidity reducer in response to ethanol (You *et al*., 2003). The results (Fig. 5A) showed no UPR activation and even indicated a significant reduction (*t*‐test, *P* < 0.05) in reporter levels after 6 h. In contrast, when the compounds that increased membrane fluidity (benzyl alcohol, tergitol, palmitoleic acid (C16:1) or DMSO) were added to the cell culture (Fig. 5B), the UPR reporter levels increased. To confirm that these UPR activations were due to fluidification effects, we combined three fluidification compounds (palmitoleic acid (Fig. 6A), benzyl alcohol (Fig. 6B) and ethanol (Fig. 6C)) with membrane fluidity reducer oleic acid and we observed the UPR reporter levels. The quantified results (Fig. 6D) revealed that oleic acid significantly diminished (*t*‐test, *P* < 0.05) the UPR activation caused by palmitoleic acid, benzyl alcohol or ethanol. These results suggest that the fluidification effect on the membrane is the origin of UPR activation upon ethanol stress. We also evaluated if the addition of these compounds, as previously described for oleic and palmitoleic acid (Thomas *et al*., 1978; You *et al*., 2003), would enhance cell growth or viability. No significant differences (*t*‐test, *P* < 0.05) were observed for our conditions (results not shown).

### Membrane fluidification mutant erg2 increases UPR activation

**Figure 7:**
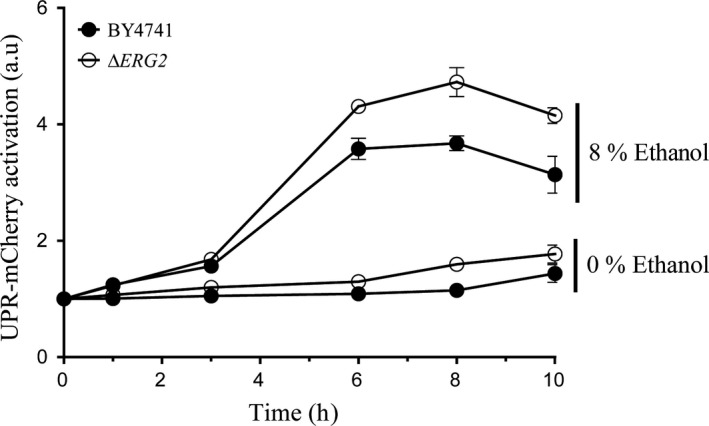
Mutant erg2 presents altered UPR activation in response to ethanol stress. The cells from BY4741 and the erg2 mutant were exposed to 8% ethanol, and samples were obtained at different time points. UPR‐mCherry reporter fluorescence was measured by flow cytometry. The results refer to time point 0 for each condition and represent the average and standard deviation of three independent biological replicates.

We followed a different approach to compare this line of thought. We evaluated UPR activation in the erg2 mutant, which shows altered membrane lipid composition and significantly increased membrane fluidity (Sharma, 2006). We evaluated the 4xUPRE‐mCherry UPR activation reporter in a plasmid in both strain BY4741 and an erg2 mutant in this background after ethanol stress. The results (Fig. 7) showed a significant increase (*t*‐test, *P* < 0.05) in UPR activation in the erg2 mutant compared to the control strain. A slight increase in UPR was observed, even in the absence of ethanol stress. These results confirm that membrane fluidification plays an important role in the UPR activation mechanism in the cellular response against ethanol stress.

## Discussion

### The UPR activation mechanism in response to ethanol stress

The results presented herein suggest that the main effect on yeast cells produced by ethanol at low physiological levels, which was an increased membrane bilayer fluidity, was the origin of UPR activation. Our results confirm previous studies, which observed changes in membrane composition to counteract fluidification after short‐term ethanol stress (Alexandre *et al*., 1994; You *et al*., 2003). Under long‐term‐adapted environmental conditions in ethanol, yeast cells seem to produce different membrane composition changes (Lahtvee *et al*., 2016). The rather slow activation of fluorescent UPR reporters, which reached maximal values at around 4–8 h, could imply that an indirect ethanol effect, such as changes in membrane composition, could activate the UPR pathway. However, the fast activation of *HAC1* mRNA 5 minutes after ethanol stress (Navarro‐Tapia *et al*., 2017), and the subsequent activation of the UPR target genes within 1 h after stress, as observed herein, ruled out this possibility, and suggested that a direct fast effect of ethanol on the yeast membrane would activate the UPR pathway. The changes in membrane composition that we observed as a result of ethanol shock suggested that they could modulate UPR activation and promote UPR signalling deactivation due to late membrane fluidity restoration and would not be the signal that fired the response. The delay between mRNA and fluorescent protein activation could merely result from protein biosynthesis difficulties and the very slow growth observed after ethanol shock.

UPR sensor Ire1 is a membrane protein designed to respond to the presence of unfolded proteins. However, the role of ethanol in generating protein denaturation at physiological concentrations is uncertain (Navarro‐Tapia *et al*., 2017). Although a certain degree of protein denaturation has been suggested at 15% of ethanol, or above (Nemzer *et al*., 2013), no protein denaturation has been described at 6% or 8% ethanol, at which we observed UPR activation (Navarro‐Tapia *et al*., 2016, 2017). These data suggest that a different signal other than protein denaturation activates UPR response at these ethanol concentrations.

We observed that several agents with demonstrated membrane fluidification properties induced the UPR pathway. One might believe that these agents could denature proteins and thus induce the UPR pathway. However, previous studies have ruled out this possibility. It has been shown that tunicamycin generates unfolded proteins, but DMSO, used as a tunicamycin solvent control, does not induce significant denaturation effects on cellular proteins at these levels (Piña and Niwa, 2015). No protein denaturing effects have been described for palmitoleic acid in yeast cells. For tergitol and benzoic acid, similar concentrations could produce a partial denaturation to specific proteins in experiments performed *in vitro* (Singh *et al*., 2010), but no direct effect has been reported *in vivo* in yeast cells. Is important to remark that, except for DMSO, no direct evidence is available to totally rule out the protein unfolding effect for these agents, thus specific studies must be conducted to demonstrate this. Nevertheless, the fact that the membrane rigidification produced by oleic acid reduced the UPR activation by these agents confirmed that its effects on the membrane induced the response rather than an effect on protein folding. A different experimental approach that used the erg2 mutant has confirmed previous suggestions.

The mechanism proposed herein for membrane changes in lipids, responsible for fluidity change as the origin of UPR activation, has been proposed in other models, such as worms (Hou *et al*., 2014) or mammalian cells (Ariyama *et al*., 2010; Koeberle *et al*., 2015), and a more general role of the UPR pathway as a membrane lipid sensor has also been proposed (Volmer *et al*., 2013). These works have demonstrated that the mutants, which present alterations in the degree of membrane lipids saturation, which changes fluidity levels, could specifically induce the UPR pathway. Our results confirm that this function of the UPR seems to be conserved between yeast and higher eukaryotes.

### UPR and Ire1 sensor: a general fluidity response pathway in yeast?

In the yeast *S. cerevisiae*, some molecular mechanisms have been proposed to play a role in sensing and responding to changes in membrane fluidity by changing membrane lipid levels, such as the lipid unsaturation ratio or PC. Regarding the lipid unsaturation ratio, several cell wall integrity (CWI) signalling pathway components have been identified in a screen for yeast deletion strains, whose growth is differentially affected by C16:1 and C18:1, supplemented in growth medium (Lockshon *et al*., 2012). Regarding PC levels, the activity of Pct1p, a membrane enzyme involved in PC biosynthesis, strongly depends on membrane insertion and binding, determined by lipid composition and activated by anionic lipids (Johnson *et al*., 1992). Therefore, low PC levels could directly activate PC biosynthesis.

The relation between the unfolded protein response (UPR) and lipid metabolism is well‐known (Cox *et al*., 1997). Several lipid biosynthetic genes, such as *INO1*, are activated by the UPR in response to inositol depletion (Travers *et al*., 2000) and, conversely, UPR‐regulated genes respond to phospholipid metabolism changes and lipid homoeostasis perturbations (Jesch *et al*., 2006; Pineau *et al*., 2009). UPR activation by lipid stress occurs independently of a functional luminal domain of Ire1, which associates with unfolded proteins (Promlek *et al*., 2011). Moreover, UPR activation by inositol starvation has been shown to occur in the absence of increasing levels of unfolded proteins (Cox *et al*., 1997; Promlek *et al*., 2011). Combined studies have indicated a separate mechanism for activating UPR in response to lipid stress, in which the cytosolic or transmembrane domain of Ire1 serves as a membrane sensor. Our data suggest that Ire1 can be activated by changes in membrane fluidity to maintain homoeostasis, as observed in the activation of the IreΔIII mutant in response to ethanol. Further work is needed to confirm that UPR signalling is a key pathway in sensing and maintaining membrane fluidity.

In conclusion, although UPR activation by ethanol shock has been recently described, its causes have not been studied in depth. Here, we show that increases in membrane fluidity could activate the UPR and give rise to further studies to reveal the role of this pathway in fluidity sensing and homoeostasis. Hence, new tools can be developed to improve ethanol tolerance in yeasts, as can biotechnological applications. The implication of this study can be important for many biotechnological applications, such as bioethanol production, but also for the production of other alcohols and compounds that affect yeast membrane fluidity by causing stress and low yields. In spite of our results, the yeasts with an enhanced UPR response could better cope with ethanol stress in these biotechnological applications. These yeasts can thus increase biomass or product yields by showing better performance when ethanol levels start to increase. Genetic modifications or natural yeast diversity screens for powered UPR responses would provide yeast with enhanced ethanol resistance and potential biotechnological applications.

## Experimental procedures

### Strains, media and culture conditions

Strain YPL004 (BY4741 Kar2‐sfGFP::HIS; UPR‐mCherry::URA, Lajoie *et al*., 2012) was used to evaluate UPR activation with fluorescent reporters. For the erg2 mutant experiments, BY4171 and BY4741Δerg2, obtained from EUROSCARF, were transformed with the pMP47 plasmid that contains the 4xUPRE‐mCherry reporter (Merksamer *et al*., 2008). KMY1015 IRE1 and KM1015 IRE1ΔIII strains, that contain UPR‐LacZ reporter, are described in Promlek *et al*. (2011).

The basal growth media selected for the experiments were standard synthetic minimal medium containing 2% glucose as a carbon source, with or without inositol (Formedium™). Media were modified whenever necessary with inositol (10, 90 or 400 μM), benzyl alcohol (20 mM), tergitol (1% v/v), DMSO (250 mM), oleic acid (650 μM), palmitoleic acid (650 μM) or ethanol (8% (v/v)). Cultures were incubated at 28°C with agitation. All the experiments were carried out with three replicates.

### Growth experiments under ethanol stress

Strains were grown overnight in minimal medium and were allowed to reach the exponential phase. Cells were washed three times with ultrapure water to remove residual media. Then the culture was divided into sterile centrifuge tubes, pelleted and incubated with different media, with or without 8% (v/v) ethanol or other compounds. Cells were grown at 28°C, sampled at different time points, pelleted and frozen in liquid nitrogen until used. Fluorescence was measured by an LSR Fortessa flow cytometer (BD Biosciences) equipped with a 488‐nm laser (525/50 bandpass filter) for sfGFP, and also with a 561‐nm laser (610/20 bandpass filter) for mCherry and the FACSDIVA software to compile.fcs files. Files were analysed by FloJo (Tree Star Ashland, OR, USA). Median fluorescence intensities (MFI) were calculated for each channel and normalized to the zero time for each condition. Biological triplicates were performed in all cases. Yeast growth curves were monitored in a SPECTROstar Omega instrument (BMG Labtech, Offenburg, Germany).

### Polar lipid determination

Cells, obtained as described above, were processed for lipid extraction. Prior to lipid extraction, a 100 ml solution of cold methanol and 20 ml of EDTA 0.1 mM were added to yeast cells with 1 g of glass beads (0.5 mm, Biospec Products, USA) in Eppendorf tubes to be mixed for 5 min in a mini‐bead‐beater‐8 (Biospec Products, Qiagen, USA). Lipid extraction was performed according to the protocol described by Redón *et al*. (2009). The lipid analysis was completed at the Kansas Lipidomics Research Center (KLRC) by an automated ESI‐MS/MS approach. A detailed protocol can be found in Singh *et al*. (2010).

### qPCR assays

The BY4741 strain was grown overnight in SC medium at 28°C and transferred to fresh medium until the log phase was reached (OD_600_ ~ 0.4). Then the culture was divided into two flasks, the second with ethanol to obtain a final concentration of 8% (v/v). Each flask was divided into three flasks to obtain three biological replicates. Next 50‐ml samples were taken at different times. Cells were quickly harvested by centrifugation, washed and frozen in liquid N_2_. RNA was extracted using the commercial NucleoSpin^®^ RNA kit (Macherey‐Nagel), according to the manufacturer's instructions. Its integrity was verified in an ND‐100 NanoDrop Spectrophotometer and by gel electrophoresis. Reverse transcription reactions were carried out using EuroScript Reverse Transcriptase (EuroGentec, Belgium), following the manufacturer's protocol.

The primers used for qPCR are described in Table 1. LightCycler^®^ 480 SYBR Green I Master (Roche) was applied for each qPCR reaction, and a PCR was run in the LightCycler^®^ 480 real‐time PCR system. All the samples were processed for the melting curve analysis, amplification efficiency and DNA concentration determinations. A mixture of all the samples was performed, and serial dilutions from the mixture (10^−1^ to 10^−5^) were used to obtain a standard curve to interpolate sample data. Two constitutive reference genes were utilized (*ACT1* and *RDN18‐1*) to normalize the amount of mRNA. These genes showed excellent uniformity at the expression levels under ethanol stress (Trotter *et al*., 2002; Penacho *et al*., 2012). To perform this normalization, a ratio was obtained between the target gene expression value and a geometric average of the *ACT1* and *RDN18*‐1 expression value. The different time points of each gene were finally referred to the time point 0 h and to the data from the control experiment with 0% ethanol.

**Table 1:** The primers used for the qPCR experiments

Primer name	Sequence
ERO1‐F	TGAAGGAGGCAGGCAAATCG
ERO1‐R	TACCGTTAGAGGGCCTTGGA
HAC1‐F	GTACAATGGAGCCTGCGACT
HAC1‐R	GGCCACCGCATCAAACAAAT
HLJ1‐F	ATTTGGGCCTTCTGCTTCCA
HLJ1‐R	TGCTTGTTGTTGCTGCTGTC
LHS1‐F	GCTCGTCAGGAGTTGCGTAT
LHS1‐R	AGTAAAAGCCAAACGGCTGC
ACT1‐F	GAAATGCAAACCGCTGCTCA
ACT1‐R	TACCGGCAGATTCCAAACCC
INO1‐F	AGAGATTGCTCCTTCCACGA
INO1‐R	ACTTGGTTTGTCCCGACTTG
RDN18‐F	TTGCGATAACGAACGAGACC
RDN18‐R	CATCGGCTTGAAACCGATAG
MPD1‐F	CCCCCAATGAGGGTCCTTTT
MPD1‐R	TCGTCGTGCTTGTTTCCTGA
IRE1‐F	AAGGCATCCGTTGTTTTGGC
IRE1‐R	AGTCAGAACCGGCGTCAAAT

## Conflict of Interest

None declared.

## Supporting information


**Fig. S1**. Yeast growth in the presence of different levels of inositol after no stress, ethanol or DTT stress.
