# Supplementary material for: Membrane fluidification by ethanol stress activates unfolded protein response in yeasts
Source: Microb Biotechnol. 2018 Feb 22;11(3):465–75. doi: 10.1111/1751-7915.13032 (PMC5902320; doi:10.1111/1751-7915.13032)
Supplement: Supplementary file 1 — Fig. S1. Yeast growth in the presence of different levels of inositol after no stress, ethanol or DTT stress. [file MBT2-11-465-s001.docx]

**Supplementary Figure 1. Yeast growth in the presence of different levels of inositol after no stress, ethanol or DTT stress**. Cells were exposed to different levels of inositol content (0, 10, 90 and 400 μM), alone or with 8% ethanol or 1mM DTT stress. The results represent the average and standard deviation of three independent biological replicates.
