# Supplementary figures and images for: Membrane fluidification by ethanol stress activates unfolded protein response in yeasts
Source: Microb Biotechnol. 2018 Feb 22;11(3):465–75. doi: 10.1111/1751-7915.13032 (PMC5902320; doi:10.1111/1751-7915.13032)

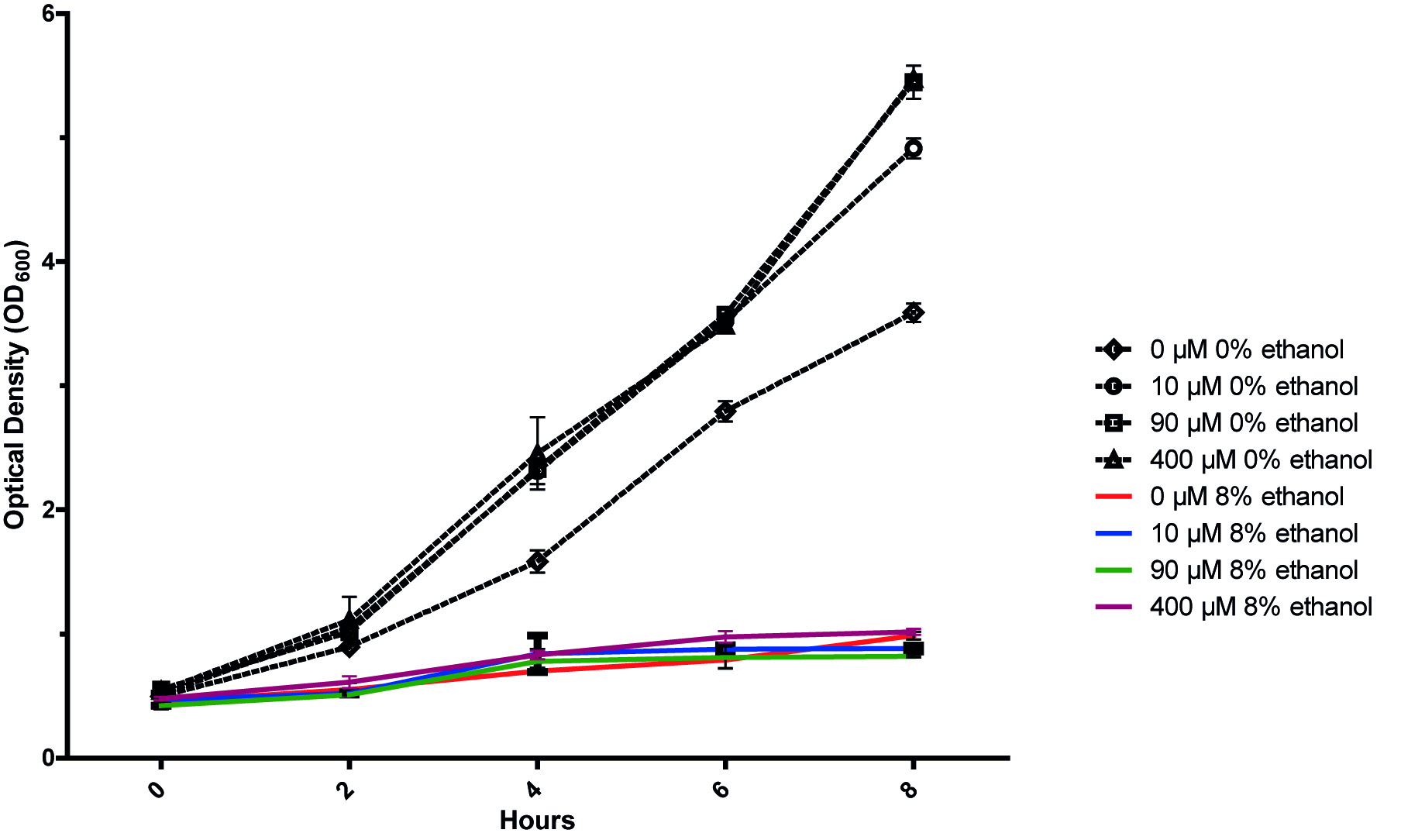

Supplement: Supplementary file 2 [file MBT2-11-465-s002.tif]
